# Assessment of Larval Toxicity and the Teratogenic Effect of Three Medicinal Plants Used in the Traditional Treatment of Urinary Tract Infections in Benin

**DOI:** 10.1155/2021/1401945

**Published:** 2021-12-07

**Authors:** Phénix Assogba, Victorien Dougnon, Edna Hounsa, Pierre Badjabaissi, Rachida Moussa Tari, Jean Robert Klotoe, Honoré Bankole, Aboudoulatif Diallo

**Affiliations:** ^1^Research Unit in Applied Microbiology and Pharmacology of Natural Substances, Research Laboratory in Applied Biology, Polytechnic School of Abomey-Calavi, University of Abomey-Calavi, Benin; ^2^Laboratory of Pharmacology and Toxicology, Faculty of Health Sciences, University of Lomé, Togo

## Abstract

**Objective:**

*Mangifera indica* Linn, *Bridelia ferruginea* Benth, and *Alstonia boonei* De Wild are three plants commonly used in the traditional treatment of urinary tract infections in Benin. This study sets out to assess the cytotoxic and teratogenic effects of extracts of these plants on *Artemia salina* larvae and hen embryos.

**Methods and Results:**

The aqueous and ethanolic extracts were obtained by maceration of the powders in solvents. Larval cytotoxicity was performed on *Artemia salina* larvae. The teratogenic effect of these plants was evaluated on chick embryos at 100 mg/kg and 300 mg/kg. The extracts were injected on the 7th and 14th days of incubation. The quality of the hatched chicks was evaluated by the Tona score followed by the hematological and the biochemical parameter assays. The extracts did not show cytotoxicity on the larvae. The eggs treated with plant extracts at 300 mg/kg significantly lowered the hatchability rate, except for the *Mangifera indica* Linn. The chicks obtained were all at the very good quality. Then, no significant variation was observed between hematological parameters except white blood cells. For the biochemical parameters, only ASAT showed some significant variations for a few extracts. It would be important to assess the genotoxicity of the plant extracts to determine more broader toxicity. These data justify the use of these medicinal plants in traditional Beninese medicine and constitute in fact a source of production of anti-infectious drugs.

## 1. Background

The use of the medicinal plants is a common practice around the world and is a part of the human culture in some parts of the planet [1]. In sub-Saharan Africa, the traditional herbal medicines are alternative to the modern chemical and the industrial drugs [2]. They are widely used in the rural and even the urban areas irrespective of gender, age, and whether heavily pregnant or not [2]. The studies have shown that most women use herbs during their pregnancy to relieve nausea and vomiting, to increase uterine tone, or to treat infections, including the candidiasis and the urinary tract infections [3]. Although, medicinal plants are natural and harmless products, they could have deleterious effects on health. Indeed, certain plants used in the traditional treatment of human pathologies can cause undesirable effects. This may include the hepatotoxicity and the teratogenic effects, especially if they are taken in excessive doses [4–6]. Thus, for the well-being of the populations, research has focused on knowledge gaps in the medicinal plants and their potential toxicities strongly encouraged by many medical organizations and by researchers in complementary and alternative medicine [3, 7]. The toxicity of a plant has been shown to depend on various factors, including the strength of secondary metabolites, the amount consumed, and the time of exposure.

In Benin, the use of medicinal plants is an essential practice of the culture and for the traditional health system. *Mangifera indica* Linn, *Bridelia ferruginea* Benth, and *Alstonia boonei* De Wild are three plants commonly used in the traditional treatment of urinary tract infections in Benin [8]. Several studies have established the toxicological profile of these plants. Multivariate toxicological studies carried out on extracts from different parts of *Mangifera indica* Linn showed that this plant is not toxic to the animals used and does not interfere with hematological and biochemical parameters. This plant also exhibited no genotoxic effects [9–11]. The works of Awodele et al. [12] showed that the aqueous extract of the stem bark of *Bridelia ferruginea* Benth did not cause mortality in rodents administered orally at various doses of 250 mg/kg to 4000 mg/kg. Regarding *Alstonia boonei* De Wild, studies carried out with extracts show that this plant species has no toxic effect at the doses tested in the models used [13–15]. It clearly appears that the toxicological studies on *in vitro* and *in vivo* models are very important before the use of medicinal plants.

From the above, it emerges from all the toxicological studies carried out on these three plants that none have addressed the teratogenic effect of these plant species.

In addition, these plants are heavily used by the pregnant women to treat bacterial infections [8]. This study evaluated the cytotoxic and teratogenic effects of aqueous and ethanolic extracts of *Mangifera indica* Linn, *Bridelia ferruginea* Benth, and *Alstonia boonei* De Wild on *Artemia salina* larvae and hen embryos.

## 2. Main Text

### 2.1. Material

The plant material consists of aqueous and ethanolic extracts of *Alstonia boonei* De Wild, *Bridelia ferruginea* Benth, and *Mangifera indica* Linn. These plants were, respectively, identified at the national herbarium of Benin (University of Abomey-Calavi) by Professor Hounnankpon Yedomonhan under the numbers YH 533/HNB, YH 534/HNB, and YH 535/HNB. The biological material was *Artemia salina* eggs (ARTEMIO JBL D-67141Gmbh Neuhofem) and chicken egg Bleu Hollandais.

### 2.2. Methods

Before the extraction, the plants were collected in the Municipal City of Lokossa, dried in the laboratory at 16°C (60.8-degree Fahrenheit) before being made into powder. For the extraction, fifty grams of the powder of each plant was macerated in 500 mL of the solvent for 72 hours. The homogenate obtained was filtered three times. This filtrate was then dried at 45°C (113-degree Fahrenheit) in an oven.

#### 2.2.1. Larval Cytotoxicity Test of Plant Extracted

The cytotoxic effect of the extracted plant was evaluated following an adaptation of the method used by Legba et al. [16]. A serial dilution of 2 in 2 was carried out from 1 mL of the stock solution of plant extract prepared at 20 mg/mL in 10 tubes. The Lethal Concentration 50 (LC_50_) was determined. The standards used to assess the cytotoxic effect of plants are presented in Table 1.

#### 2.2.2. Teratotoxicity

Bleu Hollandais brand hen eggs were purchased at Lomé (Togo). After weighing, the eggs were divided into lots (*n* = 10 eggs per lot) according to weight and then incubated in an incubator (37.7°C, 55% relative humidity, 0.06% CO_2_, and 1/60 min turning). After seven days of incubation, all the eggs were candled, and only fertile eggs were used for inoculation of the substances [17, 18]. Two concentrations were used for each plant extract: 100 mg/kg and 300 mg/kg. Into each egg, 100 *μ*l of extract was injected in the inner tube, and the pierced parts were closed with Hypafix. The batches formed are as follows:

- *Batch 1*. Control lot having received nothing
- *Batch 2*. Control batch having received only physiological water (NaCl)
- *Batch 3*. Aqueous extract of *Mangifera indica* at 100 mg/kg
- *Batch 4*. Aqueous extract of *Mangifera indica* at 300 mg/kg
- *Batch 5*. Ethanolic extract of *Mangifera indica* at 100 mg/kg
- *Batch 6*. Ethanolic extract of *Mangifera indica* at 300 mg/kg
- *Batch 7*. Aqueous extract of *Bridelia ferruginea* at 100 mg/kg
- *Batch 8*. Aqueous extract of *Bridelia ferruginea* at 300 mg/kg
- *Batch 9*. Ethanolic extract of *Bridelia ferruginea* at 100 mg/kg
- *Batch 10*. Ethanolic extract of *Bridelia ferruginea* at 300 mg/kg
- *Batch 11*. aqueous extract of *Alstonia boonei* at 100 mg/kg
- *Batch 12*. aqueous extract of *Alstonia boonei* at 300 mg/kg
- *Batch 13*. Ethanolic extract of *Alstonia boonei* at 100 mg/kg
- *Batch 14*. Ethanolic extract of *Alstonia boonei* at 300 mg/kg

Supplementary Figure S1 shows the egg weighing, the arrangement in incubator, the candling, and the inoculation of plants in the air chamber. The quality of the hatching chicks was assessed according to a descriptive scheme based on the characteristics of the hatched chick [19].  Table 2 shows the parameters evaluated with the scores for each parameter. After that, four chicks per batch were sacrificed, and then, the heart, liver, and yolk sac were removed. Blood samples were taken in EDTA tubes and dry tubes for hematological and biochemical examinations. Supplementary Figure S2 shows pictures of blood collection, dissection, and organ harvesting. The following different formulas were used to calculate the weighing parameters:

(1) $$\begin{array}{c} \text{Fertile hatching rates}=\frac{\text{Number of chicks at hatching}}{\text{Number of fertile eggs incubated}}\times 100,\\ \text{Mortality rate }\left(\text{stillbirth}\right)=\frac{\text{Number of dead chicks}}{\text{Number of fertile eggs incubated}}\times 100,\\ \text{Relative weight of organs }\left(\text{liver},\text{heart},\text{yolk sac}\right)=\frac{\text{Organ weight}}{\text{Chick weight}}\times 100,\\ \text{Relative weight of chick without yolk sac}=\frac{\text{Chick weight without bag }}{\text{Chick weight}}\times 100. \end{array}$$

### 2.3. Statistical Analysis

The GraphPad Prism version 8.0 software was used for the graph design and statistical analysis. Using ANOVA, the means and standard deviation were presented and each experimental batch was compared to the control batch for each parameter investigated by Dunnett's multiple comparisons test (ANOVA two-way). A significance level of 5% was applied for the tests performed.

## 3. Results

### 3.1. Larval Toxicity of Extracts

**Figure 1:**
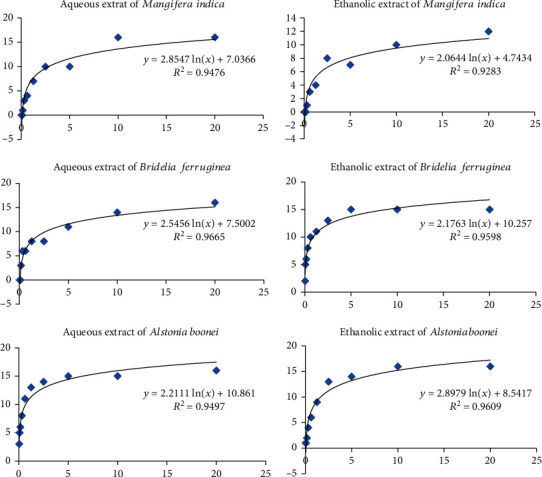
Sensitivity of *Artemia salina* larvae to aqueous and ethanolic extracts of the plants tested.

The *Artemia salina* model was used to assess the cytotoxic effect of the extracts.  Figure 1 shows the logarithmic regression curves, which express the percentage of dead larvae as a function of the concentration of the extract's plants. We recorded a decrease in surviving larvae as the concentration of extracts increased. None of the extracts showed an LC_50_ of less than 0.1 mg/mL (Table 3). All the extracts were therefore noncytotoxic at the concentration tested.

### 3.2. Effect of Extracts on Hatch Rate and Quality of Hatched Chicks

**Figure 2:**
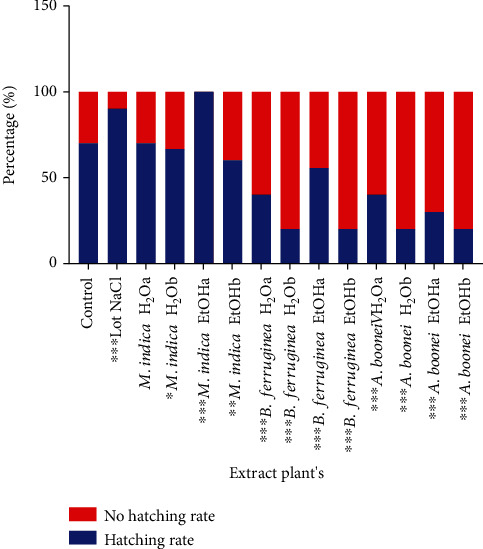
Hatch and nonhatch rates of the different study batch.

**Figure 3:**
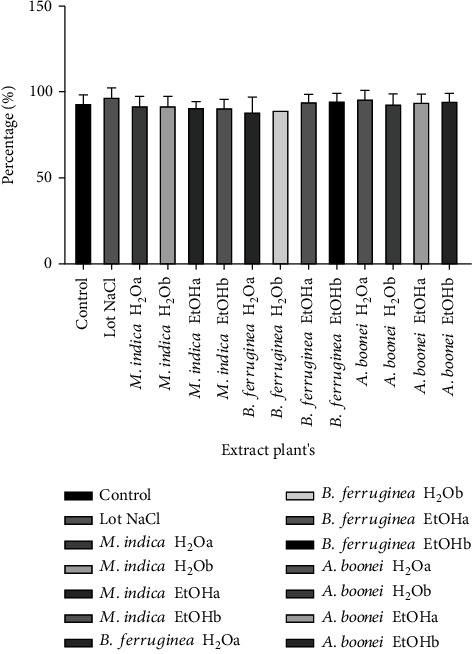
Chick quality assessed by the Tona score.

From Figure 2, it emerges that the nonhatching rates in the batches of eggs injected with the different extracts and at the various doses were significantly higher compared to those of the control batch (*p* < 0.05) except in the case of aqueous extract of *Mangifera indica* Linn at 100 mg/kg of egg weight. The injection of NaCl gave a significantly higher hatching rate than the control. The Tona score showed that all the chicks were at very good quality (Figure 3).

### 3.3. Effect of Extracts on the Weight of Chicks and Vital Organs

Chicks obtained after hatching had an average weight between 28.32 ± 0.38 g and 31.52 ± 1.65 g. The relative weights of chicks without the yolk sac were proportionally high according to the weight of the chicks (Table 4). The extracts did not cause any significant variation in these different organs compared to the respective controls (Table 5).

### 3.4. Effect of Extracts on Hematological and Biochemical Parameters

The extract did not cause any significant variation in hematological parameters except white blood cells and platelets (Table 6). In the case of biochemical parameters, no significant differences in uremia, serum creatinine, or ALAT were noted. Significant variations were noted for ASAT (Table 7).

## 4. Discussion

The objective of this study was to evaluate the cytotoxic and teratogenic effects of the extracts of *Mangifera indica* Linn, *Bridelia ferruginea* Benth, and *Alstonia boonei* De Wild on *Artemia salina* larvae and hen embryos.

From the results of larval cytotoxicity, it appears that all the extracts have an LC_50_ greater than 0.1 mg/mL, a concentration above which the extracts of medicinal plants are considered noncytotoxic. It is important to note that several studies have shown the utility and relevance of larval toxicity tests on larvae in preliminary toxicity studies [20]. Additionally, a positive correlation was even demonstrated between the larval toxicity test and the lethal oral dose of medicinal plants in mice [21].

The teratogenic effect of the aqueous and ethanolic extracts of the three plants was evaluated in Dutch Blue hen embryos. Eggs treated with the extracts exhibited reduced hatching rates due to embryonic mortalities compared to control batches, particularly batches treated with extracts prepared at 300 mg/kg. The batch treated with NaCl gave a higher hatch rate than the control batch that received nothing. All batches treated with the extracts at 300 mg/kg exhibited the lowest hatchability. These data could be explained by the fact that the injections of extract plants stopped the embryonic development of the incubating eggs. Mortalities induced by plant extracts are classified as early embryonic deaths. In fact, the heart, the first functional organ from the fourth or fifth day of incubation, could be exposed to natural substances that are herbal extracts in the case of this study. By this mechanism of embryonic development, one could deduce that the early embryonic mortalities obtained in this study would be due to the exposure of the heart to the extracts of the administered plants. An embryonic and histopathological toxicity study of *in vivo* inoculation of aflatoxin fungal extracts in chick embryos revealed high embryonic mortality rates [22]. It could be inferred that the injection of the herbal extracts used in this study on the 14th day of incubation did not have enough effect on fetal viability. The works of Ul-Hassan et al. [23] suggest that the resistance of chick embryos to toxic substances is related to the age. This hypothesis is supported by the works of Celik et al. [24], who reported that chick embryos were more sensitive to aflatoxin B1 on day 1 than on day 7 of the age. The increase in the age-related resistance of embryos to toxins is linked to the activation of the detoxification mechanism when the liver and kidneys are functional according to Khan et al. [25]. It is important to report that the batch treated with NaCl exhibited a higher hatchability rate than the control batch that received nothing. This finding shows that all embryonic mortalities would certainly not be due to extracts from the plants evaluated but probably to other factors that were not evaluated in this work. These may be, for example, genetic mutations. The chicks obtained after hatching from eggs treated with the various plant extracts were at the very good quality according to the Tona score. In addition, no apparent malformations were noted. According to the work of Tona et al. [19], the quality of the chicks can be related to the quality of the incubating eggs and the storage time of the eggs before incubation. Thus, the storage of the eggs before its incubation can deteriorate the internal quality of the eggs, particularly the height of the albumen which during incubation, the albumin proteins move in the amniotic fluid and are swallowed by the embryos which are then either digested in the intestine or transferred to the yolk sac where they can be used after hatching.

In toxicological studies, the weights of the liver, kidney, spleen, testes, heart, pancreas, brain, and tongue are very important clues used to assess the toxic effects of the substance being studied. The relative weights of the organs provide information on possible hypertrophy, atrophy, or swelling of these organs. In this study, no significant difference was noted in the weight of the chicks and the relative weight of the chicks without yolk sacs except in the case of the batch treated with the ethanolic extract of *Alstonia boonei* De Wild at 300 mg/kg. Injection of the aqueous and ethanolic extracts of *Mangifera indica* Linn, *Bridelia ferruginea* Benth, and *Astonia boonei* De Wild did not cause any significant variation between the relative weights of the liver, heart, and yolk sac compared to the respective controls. These data show that the extracts did not cause any hepatotoxic effects on the liver or disease states of these organs. The quality of the chicks from the results of the Tona score can also justify these data. Regarding hematological parameters, no significant variation was observed except white blood cells. The hematopoietic system is one of the preferred targets of toxic substances and, consequently, an important parameter of the physiology of humans and animals. This study showed that the extracts did not affect the hematopoietic system. In the case of biochemical parameters, no significant difference in uremia, serum creatinine, or ALAT was observed. Significant variations were noted for ASAT except for the NaCl batches, the aqueous extract of *Mangifera indica* Linn at 100 mg/kg, the ethanolic extract of *Bridelia ferruginea* Benth at 100 mg/kg, and the aqueous extract of *Alstonia boonei* De Wild at 100 mg/kg. Also, all ASAT values are high. This enzyme is a sensitive marker of possible tissue damage, especially the liver damage. This study did not explore the probable presence of lesions in the organs by histological sections.

### 4.1. Limitations

This study did not explore the acute and chronic toxicity of the plant extracts evaluated in this study. It would also be important to assess the genotoxicity of plant extracts to determine the toxicity of these plant species as widely as possible.

## 5. Conclusion

This study evaluated the *in vivo* toxicity of aqueous and ethanolic extracts of *Mangifera indica* Linn, *Bridelia ferruginea* Benth, and *Alstonia boonei* De Wild. The results obtained showed that the aqueous and ethanolic extracts of these three plants did not affect the survival of *Artemia salina* larvae and egg embryos at the concentrations tested. These results justify the use of these medicinal plants in the traditional treatment of the urinary tract infections in Benin. It would be important to explore the acute toxicity and genotoxicity of these plants for future studies.

## Figures and Tables

**Table 1:** Standards used to assess the cytotoxicity of plant extracts [16].

LC_50_ value	Cytotoxicity of the extract
LC_50_ ≥ 0.1 mg/mL	Nontoxic extract
0.1 mg/mL > LC_50_ ≥ 0.050 mg/mL	Low toxicity
0.050 mg/mL > LC_50_ ≥ 0.01 mg/mL	Medium toxicity
LC_50_ < 0.01 mg/mL	High toxicity

**Table 2:** Allocation of scores to the various parameters for evaluating the quality of chicks [19].

Parameters	Characteristics	Scores
Activity	Good	6
	Low	0

Down and appearances	Clean and dry	10
	Wet	8
	Dirty and wet	0

Resorption of the yolk sac	Chicks with a normal abdomen	12
	Chicks with large abdomen and fairly hard to the touch	0

Eyes	Open and shiny	16
	Open and nonshiny	8
	Closed	0

Legs	Normal legs and toes	16
	An infected leg	8
	Both infected legs	0

Umbilicus	Completely closed and clean	12
	Not completely closed and not discolored	6
	Not closed and discolored	0

Remaining membrane	No membrane	12
	Small membrane	8
	Large membrane	4
	Very large membrane	0

The yolk stop	No yolk	16
	Demands yolk	12
	Large egg yolk	8
	Very large egg yolk	0

**Table 3:** Cytotoxic effect of plant extracts on *Artemia salina* larvae.

Medicinal plants	Extract	LC_50_	*R* ^2^
*Mangifera indica* Linn	Aqueous extract	1.4	0.94
	Ethanolic extract	4.84	0.92

*Bridelia ferruginea* Benth	Aqueous extract	1.21	0.96
	Ethanolic extract	0.35	0.95

*Alstonia boonei* De Wild	Aqueous extract	0.32	0.94
	Ethanolic extract	0.82	0.96

**Table 4:** Effect of extracts of plants studied on the relative weight of chicks.

	Weight of chicks (g)	Weight of SSV chicks (g)	Relative weight of SSV chicks (%)
Control	30.02 ± 0.64	26.25 ± 0.62	87.44 ± 1.18
Lot NaCl	28.78 ± 0.68	25.42 ± 0.30	88.43 ± 1.34
*M. indica* H_2_Oa	30.66 ± 0.88	25.96 ± 0.67	84.85 ± 2.88
M. *indica* H_2_Ob	31.06 ± 1.11	27.55 ± 0.83	88.76 ± 0.91
M. *indica* EtOHa	30.88 ± 0.42	26.63 ± 0.28	86.37 ± 1.70
M. *indica* EtOHb	29.59 ± 0.36	25.72 ± 1.40	86.88 ± 4.29
*B. ferruginea* H_2_Oa	30.61 ± 1.09	27.17 ± 0.72	88.90 ± 1.89
*B. ferruginea* H_2_Ob	30.81 ± 1.39	27.69 ± 1.54	89.77 ± 1.58
*B. ferruginea* EtOHa	30.15 ± 0.38	26.81 ± 1.08	88.83 ± 2.57
*B. ferruginea* EtOHb	28.32 ± 0.38	26.03 ± 0.32	91.91 ± 0.13
*A. boonei* H_2_Oa	31.52 ± 1.65	27.57 ± 1.26	87.59 ± 1.97
*A. boonei* H_2_Ob	30.19 ± 0.34	27.65 ± 0.48	91.57 ± 1.34
*A. boonei* EtOHa	30.27 ± 0.84	26.34 ± 0.67	87.20 ± 2.95
*A. boonei* EtOHb	29.70 ± 0.49	27.53 ± 0.83	92.67 ± 1.27^∗^

Legend: H_2_Oa: aqueous extract at 100 mg/kg; H_2_Ob: aqueous extract at 300 mg/kg; EtOHa: ethanolic extract at 100 mg/kg; EtOHb: ethanolic extract at 300 mg/kg; SSV: without vitellin bag.

**Table 5:** Effect of the extracts on the relative weight of the organs of the chicks.

	Relative weight of the vitellin sac (%)	Relative weight of the heart (%)	Relative weight of the liver (%)
Control	8.41 ± 0.50	0.85 ± 0.04	2.84 ± 0.15
Lot NaCl	11.12 ± 0.15	0.75 ± 0.02	2.95 ± 0.21
*M. indica* H_2_Oa	11.24 ± 1.05	0.84 ± 0.03	2.52 ± 0.07
M. *indica* H_2_Ob	7.46 ± 0.32	0.73 ± 0.01	2.76 ± 0.10
M. *indica* EtOHa	9.56 ± 0.37	0.80 ± 0.02	2.94 ± 0.12
M. *indica* EtOHb	13.34 ± 0.38	0.73 ± 0.02	2.83 ± 0.13
*B. ferruginea* H_2_Oa	9.96 ± 1.27	0.74 ± 0.01	2.50 ± 0.10
B. *ferruginea* H_2_Ob	8.34 ± 0.66	0.74 ± 0.04	2.48 ± 0.02
B. *ferruginea* EtOHa	6.38 ± 0.53	0.95 ± 0.04	2.57 ± 0.09
B. *ferruginea* EtOHb	6.96 ± 0.19	0.88 ± 0.04	3.02 ± 0.34
*A. boonei* H_2_Oa	6.65 ± 0.34	0.88 ± 0.06	2.89 ± 0.11
*A. boonei* H_2_Ob	7.36 ± 0.09	0.77 ± 0.02	2.78 ± 0.17
A. *boonei* EtOHa	6.44 ± 0.24	0.80 ± 0.05	2.83 ± 0.06
A. *boonei* EtOHb	6.34 ± 0.13	0.77 ± 0.03	2.81 ± 0.03

Legend: H_2_Oa: aqueous extract at 100 mg/kg; H_2_Ob: aqueous extract at 300 mg/kg; EtOHa: ethanolic extract at 100 mg/kg; EtOHb: ethanolic extract at 300 mg/kg.

**Table 6:** Effect of aqueous and ethanolic plant extracts on hematological parameters of chicks.

	WB (10^3^/*μ*l)	RB (10^3^/*μ*l)	Hb (g/dl)	The (%)	MCV (fL)	MCH (Pg)	MCHC (g/dl)	PLT (10^3^/*μ*l)
Control	137.15 ± 3.41	2.15 ± 0.13	13.40 ± 0.61	29.55 ± 2.11	134.80 ± 2.78	62.50 ± 1.79	46.35 ± 1.31	72.50 ± 2.36
Lot NaCl	133.12 ± 3.35	1.73 ± 0.42	10.27 ± 2.54	25.42 ± 2.62	130.90 ± 5.81	59.72 ± 1.29	45.82 ± 1.62	58.00 ± 4.52^∗∗∗^
*M. indica* H_2_Oa	125.05 ± 0.37^∗∗∗^	2.20 ± 0.10	12.50 ± 0.17	26.85 ± 0.31	122.05 ± 2.51	56.90 ± 1.32	46.60 ± 0.11	44.00 ± 4.61^∗∗∗∗^
*M. indica* H_2_Ob	141.65 ± 5.14	1.41 ± 0.58	10.45 ± 3.43	25.55 ± 0.76	127.85 ± 2.56	59.95 ± 0.26	46.95 ± 1.12	44.00 ± 5.58^∗∗∗∗^
*M. indica* EtOHa	125.13 ± 2.62^∗∗∗∗^	2.23 ± 0.14	14.05 ± 0.95	27.95 ± 1.58	125.40 ± 0.86	62.75 ± 0.31	50.10 ± 0.57	72.50 ± 2.59
*M. indica* EtOHb	143.51 ± 5.31	2.17 ± 0.12	13.05 ± 0.43	25.90 ± 0.70	119.85 ± 3.20	60.15 ± 1.35	50.20 ± 0.23	60.50 ± 4.33
*B. ferruginea* H_2_Oa	130.55 ± 5.34	1.95 ± 0.09	12.35 ± 0.14	24.80 ± 0.11	127.20 ± 0.05	63.35 ± 0.95	49.80 ± 0.75	73.00 ± 1.73
*B. ferruginea* H_2_Ob	131.30 ± 2.36	2.22 ± 0.02	13.23 ± 0.18	28.25 ± 0.98	125.77 ± 3.34	59.37 ± 0.60	47.27 ± 1.07	54.50 ± 2.46^∗^
*B. ferruginea* EtOHa	139.55 ± 0.15	2.20 ± 0.13	13.25 ± 0.66	27.80 ± 1.61	126.40 ± 0.49	60.35 ± 0.77	47.75 ± 0.43	70.00 ± 3.46
*B. ferruginea* EtOHb	127.40 ± 2.15	2.24 ± 0.50	13.05 ± 0.47	28.27 ± 1.26	125.70 ± 3.00	58.12 ± 1.77	46.25 ± 0.42	50.25 ± 5.20^∗∗^
*A. boonei* H_2_Oa	115.12 ± 3.90^∗∗^	1.97 ± 0.10	12.30 ± 0.80	25.32 ± 1.73	126.72 ± 2.03	62.32 ± 0.99	41.70 ± 7.88	52.75 ± 6.67^∗^
*A. boonei* H_2_Ob	125.50 ± 0.37^∗^	2.07 ± 0.05	12.65 ± 0.08	26.20 ± 0.69	126.50 ± 0.05	61.40 ± 1.27	48.55 ± 1.01	49.00 ± 1.73^∗∗^
*A. boonei* EtOHa	126.17 ± 1.44	2.23 ± 0.02	13.20 ± 0.45	27.62 ± 0.52	124.47 ± 3.21	58.92 ± 1.82	47.32 ± 0.43	49.75 ± 6.90^∗∗^
A. *boonei* EtOHb	128.05 ± 1.35	2.23 ± 0.03	13.15 ± 0.20	27.70 ± 0.17	123.83 ± 1.47	58.80 ± 0.23	47.50 ± 2.59	47.50 ± 2.59^∗∗∗^

Legend: GB: white blood cells; NR: red blood cells; Hb: hemoglobin; Hte: hematocrit; MCV: average globular volume; MCH: average corpuscular hemoglobin content; MCHC: mean corpuscular hemoglobin concentration; PLT: platelets; H_2_Oa: aqueous extract at 100 mg/kg; H_2_Ob: aqueous extract at 300 mg/kg; EtOHa: ethanolic extract at 100 mg/kg; EtOHb: ethanolic extract at 300 mg/kg.

**Table 7:** Effect of aqueous and ethanolic plant extracts on biochemical parameters.

	Urea (g/l)	Creat (mg/mL)	ALAT (UI/L)	ASAT (UI/L)
Control	0.29 ± 0.06	8.60 ± 1.05	8.00 ± 0.91	217.50 ± 8.70
Lot NaCl	0.23 ± 0.04	7.24 ± 0.21	10.00 ± 0.40	222.00 ± 13.36
*M. indica* H_2_Oa	0.20 ± 0.02	6.73 ± 0.66	12.75 ± 1.79	220.50 ± 8.31
M. *indica* H_2_Ob	0.20 ± 0.04	7.90 ± 0.23	13.50 ± 1.3	200.25 ± 8.11^∗^
M. *indica* EtOHa	0.23 ± 0.02	7.37 ± 0.45	7.37 ± 0.45	283.50 ± 9.11^∗∗∗∗^
M. *indica* EtOHb	0.19 ± 0.02	7.89 ± 0.26	7.89 ± 0.26	195.25 ± 9.04^∗∗^
*B. ferruginea* H_2_Oa	0.20 ± 0.03	7.39 ± 0.30	12.80 ± 1.25	252.00 ± 17.92^∗∗∗∗^
B. *ferruginea* H_2_Ob	0.19 ± 0.02	8.25 ± 0.45	15.50 ± 1.32	197.00 ± 5.84^∗∗^
B. *ferruginea* EtOHa	0.20 ± 0.03	10.08 ± 0.23	7.00 ± 0.40	230.25 ± 23.00
B. *ferruginea* EtOHb	0.21 ± 0.02	9.16 ± 0.16	7.50 ± 0.86	248.25 ± 11.85^∗∗∗∗^
*A. boonei* H_2_Oa	0.22 ± 0.03	8.61 ± 0.21	7.00 ± 0.42	232.00 ± 9.37
*A. boonei* H_2_Ob	0.23 ± 0.01	9.70 ± 0.30	7.50 ± 0.64	265.00 ± 5.77^∗∗∗∗^
A. *boonei* EtOHa	0.40 ± 0.05	11.26 ± 0.29	5.75 ± 1.10	245.50 ± 8.27^∗∗∗∗^
A. *boonei* EtOHb	0.28 ± 0.03	11.30 ± 0.43	8.00 ± 0.91	256.00 ± 2.30^∗∗∗∗^

Legend: H_2_Oa: aqueous extract at 100 mg/kg; H_2_Ob: aqueous extract at 300 mg/kg; EtOHa: ethanolic extract at 100 mg/kg; EtOHb: ethanolic extract at 300 mg/kg.

## Data Availability

All the data are available upon request.

## Supplementary Materials

Supplementary Materials The supplementary figures show some images of the essential phases of the work in the laboratory. The first supplementary figure shows the images of the weighing of the chicken eggs, the arrangement of the eggs in the incubator, the candling, and the inoculation of the plant extracts in the inner tube. The second supplementary figure showed the technique of blood sampling, dissection, and organ removal.
