# Supplementary material for: Assessment of Larval Toxicity and the Teratogenic Effect of Three Medicinal Plants Used in the Traditional Treatment of Urinary Tract Infections in Benin
Source: Biomed Res Int. 2021 Dec 7;2021:1401945. doi: 10.1155/2021/1401945 (PMC8670930; doi:10.1155/2021/1401945)
Supplement: Supplementary Materials — The supplementary figures show some images of the essential phases of the work in the laboratory. The first supplementary figure shows the images of the weighing of the chicken eggs, the arrangement of the eggs in the incubator, the candling, and the inoculation of the plant extracts in the inner tube. The second supplementary figure showed the technique of blood sampling, dissection, and organ removal. [file 1401945.f1.zip › Supplementary file S1-2.docx]

**Supplementary file S1**


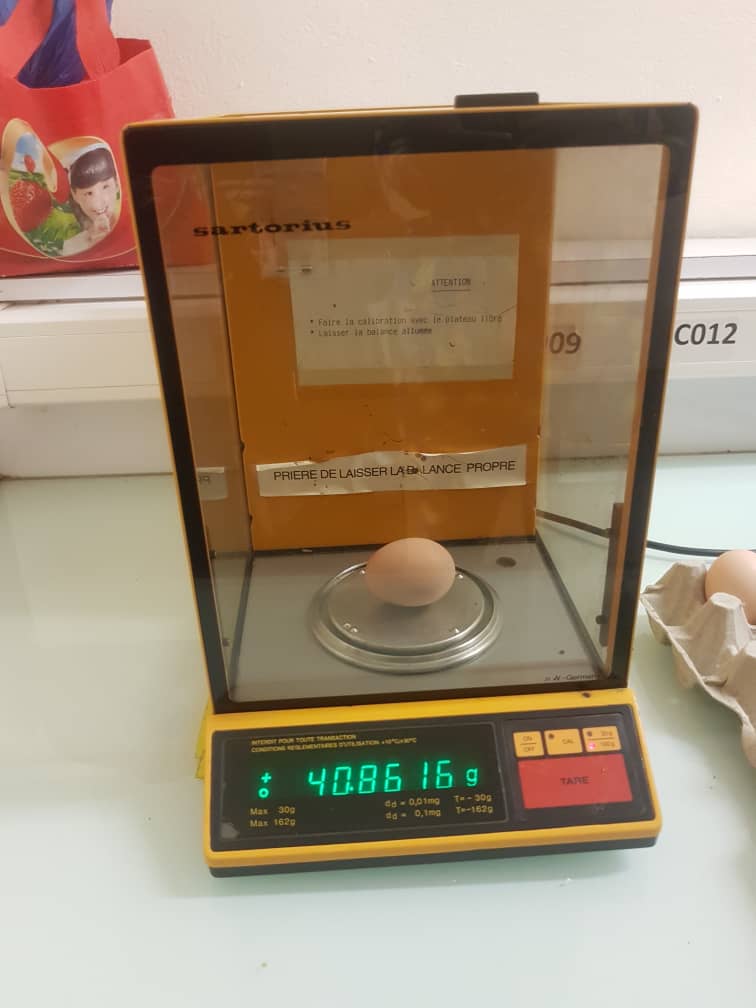

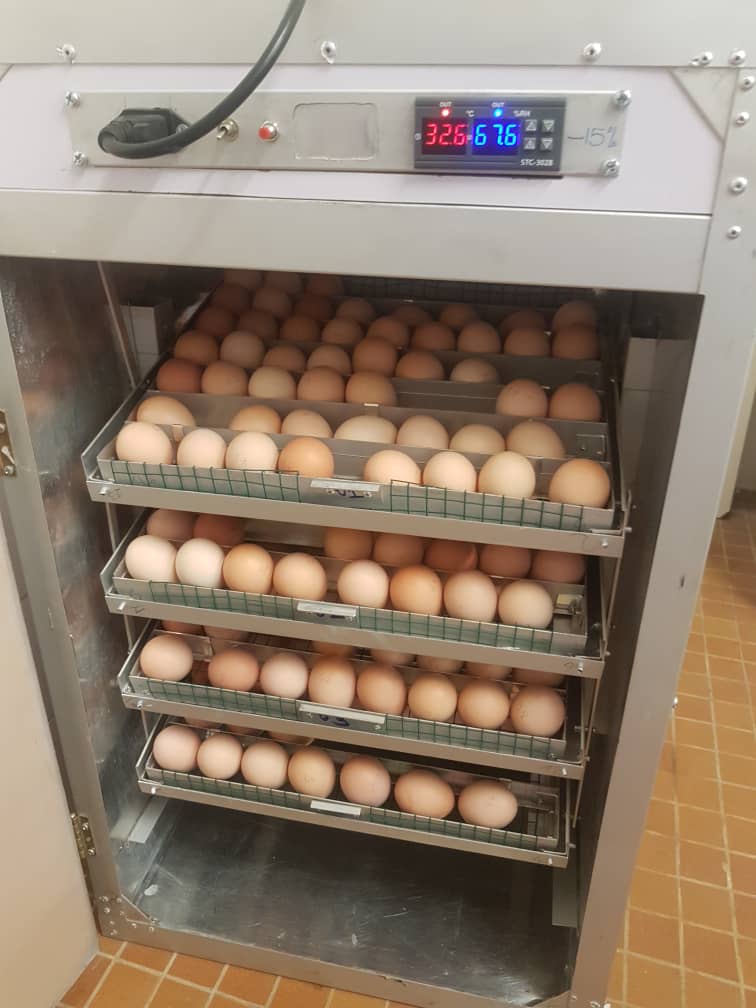


**Figure S1a :** Weighing eggs

**Figure S1b :** Arrangement of the eggs in the incubator


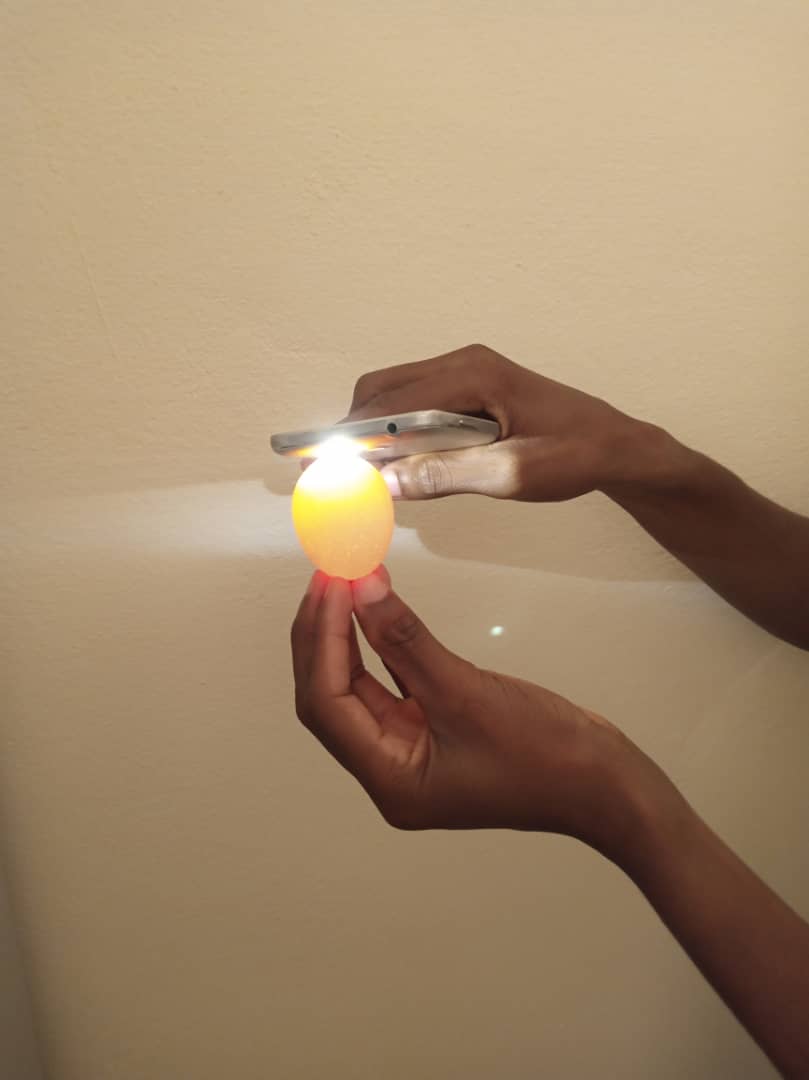

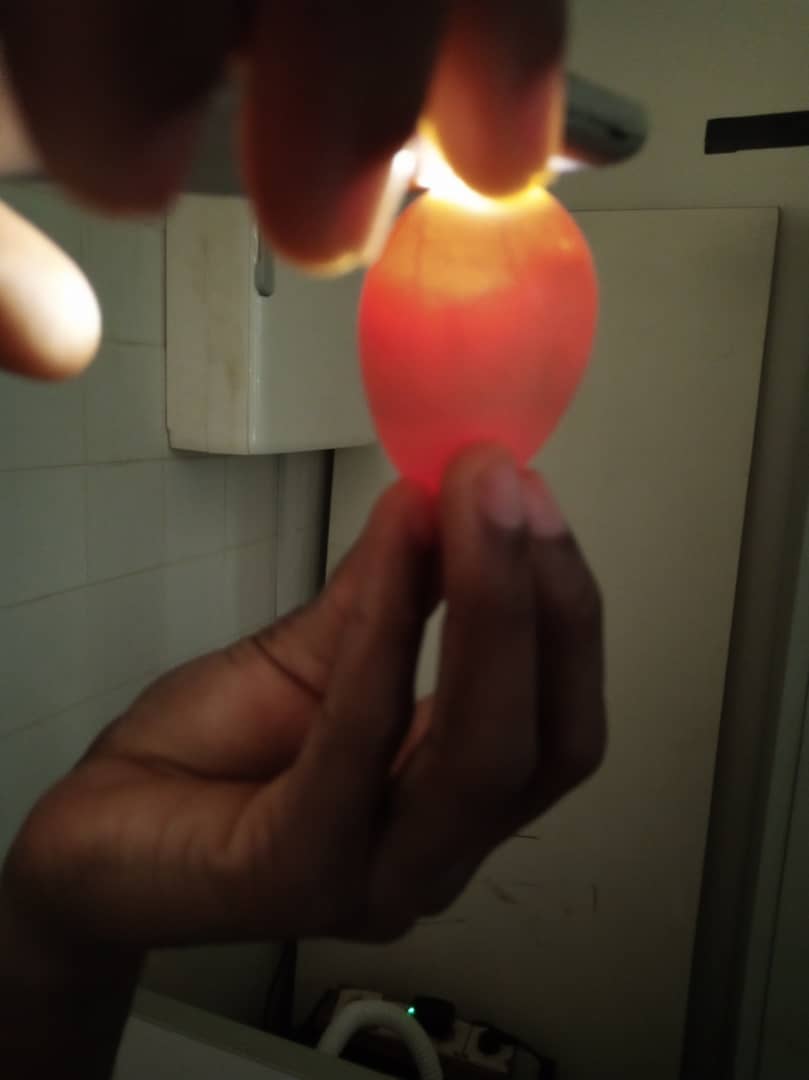


**Figure S1c :** Mirage : Unfertile egg

**Figure S1d :** Mirage : Fertile egg


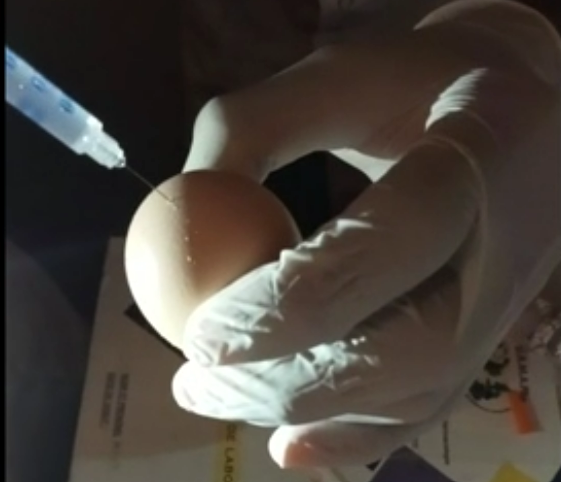

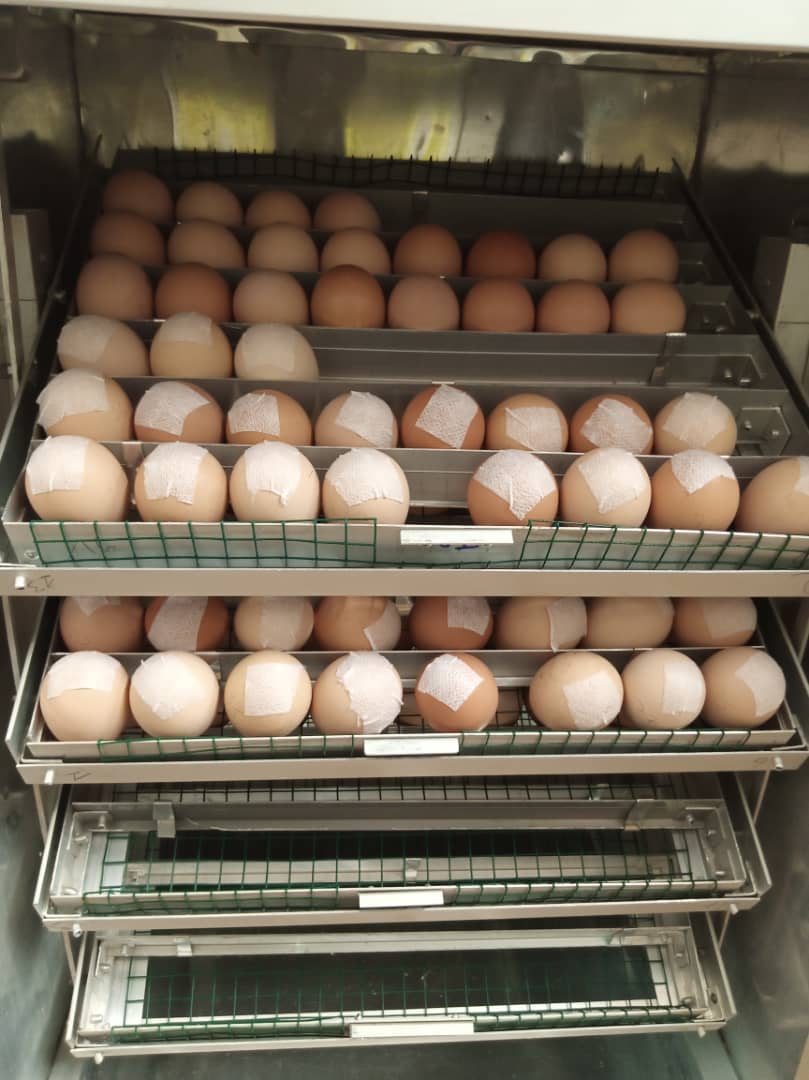


**Figure S1e :** Injection into the air chamber

**Figure S1f :** Arrangement of the eggs after injection in the incubator

**Figure S1 :** Weighing, mirage, injecting and placing the eggs in the incubator
