# Supplementary material for: Assessment of Larval Toxicity and the Teratogenic Effect of Three Medicinal Plants Used in the Traditional Treatment of Urinary Tract Infections in Benin
Source: Biomed Res Int. 2021 Dec 7;2021:1401945. doi: 10.1155/2021/1401945 (PMC8670930; doi:10.1155/2021/1401945)
Supplement: Supplementary Materials — The supplementary figures show some images of the essential phases of the work in the laboratory. The first supplementary figure shows the images of the weighing of the chicken eggs, the arrangement of the eggs in the incubator, the candling, and the inoculation of the plant extracts in the inner tube. The second supplementary figure showed the technique of blood sampling, dissection, and organ removal. [file 1401945.f1.zip › Supplementary file S2.docx]

**Supplementary file S2**


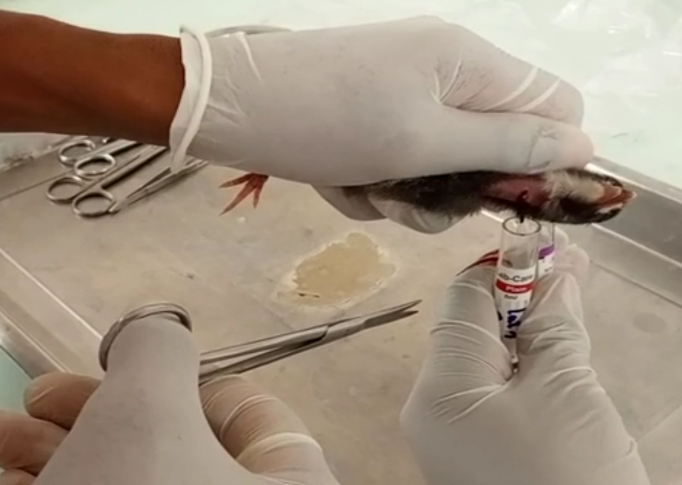

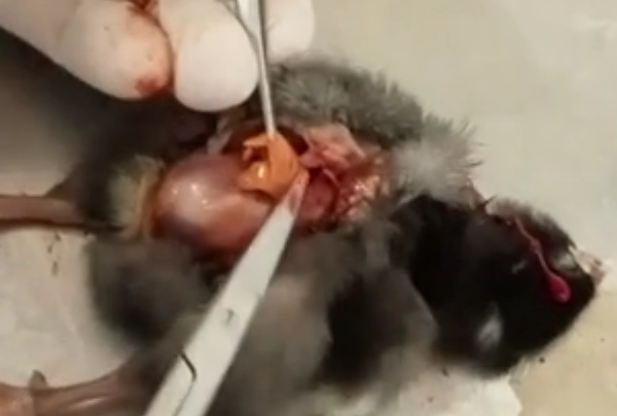


**Figure S2a :** Blood sampling

**Figure S2b:** Dissection and organs removal

**Figure S2 :** Harvesting, dissection and removal of organs from chicks
